# Swedish health professional's perceptions of Interplay, a digital support tool for parents' couple relationship and parenting

**DOI:** 10.3389/fdgth.2026.1912924

**Published:** 2026-08-05

**Authors:** Angelica Holst, Caroline Bäckström, Henrik Engström, Margaretha Larsson, Frida Lygnegård

**Affiliations:** 1 Faculty of Caring Science, Work Life and Social Welfare, University of Borås, Borås, Sweden; 2 Region Västra Götaland, Regionhälsan, Youth Guidance Centre, Falköping, Sweden; 3 Department of Caring Science, University of Borås, Borås, Sweden; 4 School of Informatics, University of Skövde, Skövde, Sweden; 5 School of Health Sciences, University of Skövde, Skövde, Sweden; 6 Department of Rehabilitation, CHILD, School of Health and Welfare, Jönköping University, Jönköping, Sweden

**Keywords:** childbirth, communication, health professionals, interpersonal relations, parenting, parents, pregnancy, support

## Abstract

**Introduction:**

Professional support is known to promote parents' transition to parenthood, however, there is a need for continued development in parental support to meet parents' needs and support them in their couple relationship. In Scandinavia a digital parental support tool, Interplay, has been developed for parents to use in their relationship during the transition to parenthood. However, research is needed on how a digital support tool such as Interplay can be included in care and professional support for parents. Therefore, the current study aims to explore how professionals perceive Interplay as a digital support tool for parents' couple relationship and parenting.

**Methods:**

Semi-structured interviews were conducted with 15 professionals who had used Interplay. The professionals were working with parental support in Swedish antenatal care, child health care and family counselling. Data were analyzed using phenomenographic analysis method.

**Results:**

The results are presented in two descriptive categories: *A complementary interactive parental support tool* and *May facilitate parental transition and be valuable to include in care*.

**Conclusion:**

Interplay has the potential to be a valuable complement to existing parental support, as it could function as a modern tool that can be used by both parents together in their everyday lives to communicate on topics related to their relationship and parenting. It would be valuable to include Interplay in care as it could be combined with professional expertise, which may lead to enhanced parental support.

## 1 Introduction

The transition to parenthood can be facilitated by care and professional support (1, 2). However, in order to meet parents' needs for support, further development in parental support is required (2). Care can be understood as a professional approach that is open to people's unique life world (3), whereby the person's individual support needs to be met for the care to be caring (4). Professional support can be provided in various forms, such as, informational, emotional and instrumental support, which can further be described as an interactive process that strengthens the person's ability to cope with life's challenges, such as giving birth to a child and becoming a parent (5). Professional support in connection with the transition to parenthood differs globally (6). The term *health professional* in this study refers to midwives, specialist nurses and family counsellors. This since parental support in Sweden is offered in several arenas, such as through midwives in antenatal care during pregnancy and the first period after the birth of the child, through specialist nurses in child health care during the first six years of a child's life (2), and through municipal family counsellors with basic psychotherapy training who offer support to couples and families throughout lives (7).

Parental support in Sweden is based on the nationally legislated convention on children's rights (8), defined as targeted interventions and activities for parents which aim to strengthen parents' competence and the relationship between parent and child. This includes supporting parents in their knowledge of the child's rights, health and development, strengthening parents' relationships and their social networks (2). Parents' couple relationship can be understood as a central part of the health of the entire family, as the quality of the relationship can affect parental behaviors and thus also the child's upbringing environment (9, 10). Recent research has shown that the quality of parents' couple relationships tends to decline after the birth of the first child and the following eight years, and that there may be a relationship between parents' estimated quality of the couple relationship and their sense of coherence and perceived social support (11). Similarly, other recent research has examined parents' decreased relationship satisfaction in connection with parenthood, showing a relationship with decreased emotional intimacy and appreciation, as well as an association with increased relationship conflict among parents (12).

In order to meet parents' needs for support in their couple relationship, there is thus a need for continued development of parental support, as well as a need to make parental support more accessible and develop more forms of support so that parents can be reached to a greater extent (2). For example, apps can be an appealing way to give parents easy access to support that can promote the health of both parents and children (13). Recent review has shown that few apps tend to be aimed at supporting parents' couple relationships and that there is a need for digital forms of support to be included in healthcare so that professionals can meet parents' needs and support them in their use of apps (14). In connection with the digitalization of society, the commercial market for apps is growing rapidly (15). Whereby professionals express difficulties in supporting parents in their use of apps due to challenges in knowing which apps are reliable and in line with healthcare recommendations (16). There is thus a need for further research to increase knowledge about digital forms of parental support and how they could be included in healthcare (17).

A digital parental support, *Interplay* (in Swedish: *Samspel*), was developed in Scandinavia for parents to use together in their relationship in connection with becoming parents. Interplay was developed as a quiz-based serious game in app format, for parents to play together in their relationship, and it aims to encourage parents to mutually discuss and reflect on issues related to their relationship and shared parenting. The purpose of Interplay is thus to facilitate parents' understanding and communication about the changes that becoming a parent means in relation to their relationship and shared parenting. This may in the long term promote parents' relationship and thereby also a safe upbringing environment for their children (18), further description of Interplay is given below.

Previous research has shown that parents perceived Interplay as a catalyst for individual reflection as well as joint reflection and communication within the couple relationship. Furthermore, Interplay provided support for the parents' ability to handle the changes that parenthood entailed, which strengthened their relationship and shared parenting (19). The term *parent* refers in this study to parents undergoing parental transition which means that they are expecting a child or have had a child within the last year. To increase understanding and knowledge on how a digital parental support tool as Interplay can be included in the care and professional support to parents during parental transition, more research is needed on professionals' experiences. Hence, the current study aims to explore how professionals perceive Interplay as a digital support tool for parents couple relationship and parenting.

## 2 Methods

Current study is part of a research project registered in ISRCTN with ID: ISRCTN18017741 http://www.isrctn.com/ISRCTN18017741, this study constitutes Study C in the study protocol which further describes the research project (18). In this study a qualitative inductive design with a phenomenographic approach has been used to explore professionals' perceptions of Interplay as a digital support tool in relation to parents couple relationship and parenting. Through a qualitative design, lived experiences can be made visible (20), whereupon people's individual experience-based and relational perceptions can be in focus through a phenomenographic approach (21), which allows for variations, similarities and differences in perceptions (22). In current study, health professionals were interviewed on their perceptions after using Interplay, which means that they played Interplay together with their own chosen partner. Some of the participants chose to play together with a colleague, while others played with a private friend or partner.

### 2.1 Interplay—A digital parental support tool

**Figure 1:**
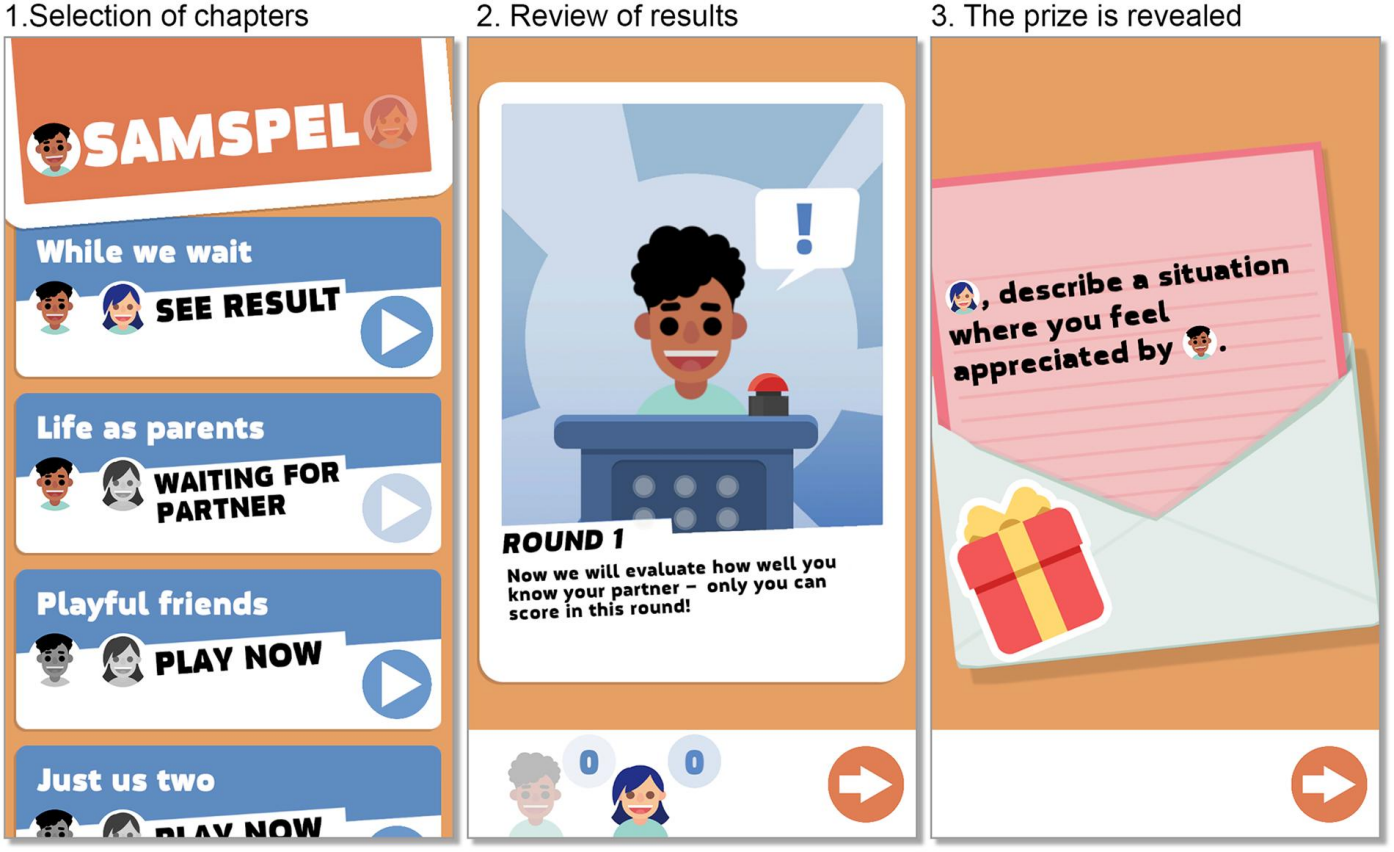
Three example screenshots from Interplay (in translation from Swedish). Each partner has selected an avatar icon that is used to represent them in all parts of the game.

Interplay has been developed through a collaboration between midwives, psychologists and a game company in Denmark. In the larger research project (18) Interplay has been translated into Swedish and Swedish contexts. Interplay is a quiz-based serious game in app format where the competition aims to be a catalyst for social interaction. While playing, parents get to touch on various topics related to the couple's relationship and shared parenting. Interplay is designed to be played between parental couples, whereby the app format aims to promote the use of Interplay regardless of time and place. Each player answers questions individually on their own smartphone. There are 15 chapters in the game which can be selected from the main screen (Figure 1, left), for example; *Life as parents, Everyday life, Under pressure, Sexytime* and *What we dream about*. Each chapter consists of three sections: their own perspective, the partner's assumed perspective and trivia questions.

The questions in each chapter are paired: partner A answers a question about themselves, while partner B guesses how partner A will respond to that question. This means every question has a correct answer, which both partners discover when they review their results together (Figure 1, middle). The result review shows both partners' answers side by side, giving them a chance to discuss how well they know each other. The player who has collected the most points receives a prize. An example of such a prize is shown in Figure 1 (right). The competitive element aims to be a catalyst for social interaction between the two partners.

### 2.2 Settings and participants

This study was conducted in southwestern Sweden in an area consisting of both urban and rural areas and with approximately 269,000 inhabitants (23). Annually, approximately 2,300 children are born in the area, which is cared for by one hospital, 15 midwife clinics, 26 child health care centres and a family counselling service. Professionals active in antenatal care (midwives) and child health care (specialist nurses in child health), as well as family counselling (family counsellors) were asked about participation in the study through convenience sampling, which can be suitable for recruiting specific professions (24). Unit managers in the area received information about the study via e-mail and asked professionals who met the inclusion criteria about participation in the study. Total 15 participants showed interest, all met the inclusion criteria and gave their informed consent to participate (six midwives, five specialist nurses, and four family counsellors), all participants completed the study. Their age was 32–65 years, the mean age 44.8 years (SD = 10.0) and they had work experience from their current profession between 1.5 and 30 years, with a mean experience of 9.2 years (SD = 8.2).

#### 2.2.1 Inclusion criteria

Midwife, specialist nurse or family counsellor active in antenatal care, child health care, or family counselling in the south-west of Sweden.

### 2.3 Data collection

Prior to data collection, a semi-structured interview guide (Supplementary Additional File 1), was developed, which is preferred within the phenomenographic tradition (22). Starting with open questions such as: *Tell us about your experiences using Interplay, how appealing did you perceive Interplay?* With the possibility of follow-up questions: *Can you tell us more? Can you give an example?* A pilot interview was then conducted to test the interview guide, the informants' understanding of the interview questions and the ability of the questions to answer the purpose of the study. The results of the pilot interview showed that the interview questions were easy to understand and no changes to the interview guide were made. The pilot interview was not included in data analysis, as the informant did not meet the inclusion criteria for the study. Participants were given the opportunity to choose the time of the interview and were encouraged to download Interplay at least two weeks in advance. The interviews were conducted by two of the authors (C.B., M.L.) digitally via Zoom during 2020–2024 and lasted between 20 and 60 min. Two participants took part in an individual interview, all other participants participated in focus group interviews. In total, four focus group interviews were conducted, the groups were profession-homogeneous with 2–4 participants. The interviews were audio recorded and transcribed verbatim. Within the phenomenographic method, a series of standard research methods can be used, such as semi-structured interviews and focus groups for transcriptions (25). In the transcription for the current study, individual participant statements could be distinguished and the analysis was based on an individual level.

### 2.4 Data analysis

Data analysis followed the phenomenographic analysis process described by Sjöström and Dahlgren (21). *Familiarisation;* To familiarize with the professionals' perceptions, the manuscript was read several times*, Compilation*; Different perceptions were identified and transferred to the data analysis software NVivo 13, *Condensation*; To create a clearer overview, perceptions were condensed to their meaning and essence, *Grouping*; Similar perceptions were grouped together, *Comparison* of *categories*; The categories of the groups were compared with each other and the transcripts*, Naming* and *Contrastive comparison of categories;* The categories were named based on the distinct cores of their meaning. The analysis process was carried out by the first author during continuous discussion with the other authors, all of whom have broad experience in qualitative methods. All authors agreed on the results of the analysis process. The authors represent both male and female perspectives with a range of professional backgrounds representing midwife, specialist nurse, occupational therapist and professor of game development, of which one of the authors is a PhD student, one is assistant professor, two is associate professors and one is a professor.

## 3 Results

The data analysis resulted in two descriptive categories: *A complementary interactive parental support tool* and *May facilitate parental transition and be valuable to include in care.* The descriptive categories with related perceptions are presented in Table 1. The results are exemplified by quotes from health professionals, translated to English and slightly edited for clarity.

**Table 1:** Overview of descriptive categories and perceptions.

Descriptive categories	Perceptions
A complementary interactive parental support tool	A modern parental support for parents to use together
	Encourages communication between parents
May facilitate parental transition and be valuable to include in care	Highlights valuable topics that may promote parental reflection and learning
	Interplay and professional competence may be combined and reinforce each other

### 3.1 A complementary interactive parental support tool

Professionals perceived Interplay as a tool that could function as a complement to existing forms of parental support. This by being able to provide a communication support for parents to use in their everyday lives that interactively includes both parents and engages them in the same process with a focus on the couple relationship and shared parenting.

#### 3.1.1 A modern parental support for parents to use together

Interplay was perceived as a unique and modern support tool that includes both parents in a couple relationship and that could complement existing parental support. Professionals believed that the digital app format could help meet parents' varied needs for flexible and digital forms of support. This since parents do not need to be dependent on specific places, times or professional contexts to be able to take advantage of the support. Instead, they may flexibly adapt the use of individual circumstances and needs in their everyday lives. However, it would not be able to function as a support for all parents as it requires parents to have access to a smartphone. There were also perceptions among professionals that it would be valuable if it included stepfamilies and separated parents to a greater extent, as parents' life situations may differ. With such functions included in the support tool, it could become more inclusive and provide support for a broader target group of parents. Likewise, it was described that parents' language skills could lead to varied conditions for using Interplay and entail risks of misunderstandings or unintentional interpretations. On the other hand, Interplay was perceived by professionals as easy to navigate, with a clear structure that could guide parents in their use.

Almost impossible to make a mistake, you were guided through the game in a playful and good way, not a lot of informational text but nice simple buttons to press. (Midwife, Antenatal Care)

Furthermore, Interplay was described as able to contribute to continuity by constituting parental support that extends from pregnancy to the time after the birth of the child. Through its format, Interplay was perceived to differ from existing forms of parental support. Professionals described that the forms of support that they are able to offer parents today often tend to consist of a more informative nature, for example through websites. Interplay was perceived as unique parental support by constituting interactive support that both parents can be involved in together. “*It is so fun that there is something digital that both can do together”* (Specialist nurse, Child Health Care). Interplay was described as a unique tool that could support parents in taking time for each other and focusing on the shared relationship, a platform for mutual participation where both parents could be involved in the same process. It could thus constitute a modern and complementary parental support that parents could use in everyday life to work together on their shared relationship and parenting.

#### 3.1.2 Encourages communication between parents

Interplay was described as a pleasurable and fun tool, and participants described that the joy of playing was perceived as an important factor in motivating parents to use it. For example, perceptions emerged that Interplay offers a varied balance of humor and seriousness, which may promote parents' joy of playing. It contained playful and engaging elements that may encourage parents to talk to each other. Similarly, the elements in Interplay were described as able to promote positive emotional experiences and a more relaxed conversational climate, which could facilitate dialogues between parents and in the long term have the potential to contribute to promoting parents' motivation for sustained use in everyday life.

…it was fun and a bit lustful. I think that was quite important so that it doesn't become so heavy. (Specialist nurse, Child Health Care)

…it was fun while at the same time there was an opportunity to put it on a serious level and continue the conversation … (Family counsellor, Family Counselling)

However, varying perceptions emerged related to parents' ability to communicate with each other. Some professionals expressed concerns that Interplay might primarily attract parents with good communication skills, while others expressed concerns that encouraging communication might not be solely of a positive nature and that there might be a risk that conflicts within couples who have existing problems in their relationship might escalate. In contrast professionals described that Interplay may function as a neutral basis for parents to approach topics that may be experienced as more difficult or sensitive to talk about.

It's good to be able to talk about certain things that may feel uncomfortable … it's brought up in a way that makes it a little easier to talk about it … it kind of comes into the game, then it becomes much easier, like neutral ground. (Midwife, Antenatal Care)

Similarly, Interplay could guide parents in how a process of storytelling, listening and subsequent confirmation could take place within the couple. In this way, professionals believed that Interplay may provide a communication support that could promote balanced conversations within the parents' relationship, which further may strengthen their ability to communicate with each other. Similarly, family counsellors described that parents often turn to them when problems have arisen in their relationship and that Interplay may function as complementary support for preventive purposes, as well as support for parents who do not want to seek professional help, by creating space and opportunities for parents to highlight and identify challenges and make possible needs for additional support visible.

### 3.2 May facilitate parental transition and be valuable to include in care

Results illustrated that Interplay could provide a support that draws attention to topics that may be relevant to parents' current life situation and the changes that becoming a parent can bring. Through this, professionals described that parents' reflections on their relationship and parenthood may be promoted, which in the long term may promote parents' learning and development in connection with the transition to parenthood. It was also perceived that it could be valuable to include Interplay in care, as Interplay was perceived to be able to be combined with professional competence, which may lead to a synergistic reinforcement of each other and thus an enhanced parental support.

#### 3.2.1 Highlights valuable topics that may promote parental reflection and learning

Interplay was described as able to provide a support that could direct parents' focus and make them aware of topics that may be valuable to address in connection with parenthood. The variety of topics in Interplay was perceived to be well in line with what professionals highlight in connection with their care of parents, as well as with their experiences of what parents need to address in relation to their relationship and shared parenthood. Some of the included topics that professionals highlighted as particularly valuable for parents to address were, for example, sex life, family formation, upbringing, caring for a sick child, parental leave, work and support from the environment. Although the results illustrated similarities with the topics that professionals raise in connection with caregiving, Interplay was perceived to have the potential to bring topics closer to parents and integrate them into parents' everyday lives. Similarly, it emerged that Interplay could provide support for highlighting topics that parents have not previously reflected on, which could promote parents' opportunities to touch on topics that are less common in their everyday conversations with each other.

…above all, I think it can help open up questions that are not natural for everyone to bring up, for example at the dinner table … (Midwife, Antenatal Care)

Furthermore, Interplay may function as a neutral basis for parents to touch on topics that may be perceived as more sensitive or difficult to talk about. Professionals described that Interplay could provide support for parents as they do not need to raise the topic themselves but rather it is part of the game. Furthermore, it was described as valuable that some topics were perceived to be characterized by humor while others by more seriousness, as the variation could promote parents' motivation and opportunities to influence direction. It was also perceived that the variation in topics could contribute to meeting parents' individual needs as the pregnancy and parents' transition to parenthood progresses. Professionals perceived this may support and strengthen parents in both their preparations and in their ability to handle the changes that parenthood entails.

…I think that Interplay would be a great alternative … a natural basis for addressing these questions that you know can be difficult to start thinking about during pregnancy …  I think that makes it much easier when you are standing there … Interplay could be a good basis for discussion to address these questions with each other because you don't know what to discuss with each other before you have any concrete questions to talk about, since you don't always know what parenthood means …  (Midwife, Antenatal Care)

Interplay was described as a parental support that may encourage parents to reflect on issues related to their relationship and parenthood. The participants perceived that Interplay could constitute a support that may facilitate for parents to imagining different scenarios that parenthood may entail. Which further may broaden parents' perspectives and promote individual as well as joint reflection within the relationship. Similarly, it emerged that Interplay may promote parents' learning and understanding of each other within the relationship, which in the long term could have the potential to promote realistic expectations and safety in the parental roles as well as in the joint relationship. Professionals drew parallels to their experiences of caring for parents and suggested that parents can gain a better understanding of each other through knowledge and communication, which can strengthen parents both as individuals and as couples and make it easier for them to handle the challenges that parenthood can bring.

#### 3.2.2 Interplay and professional competence may be combined and reinforce each other

Results illustrated that it could be valuable to include Interplay in care and that it could be combined with professional competence which in synergy could reinforce each other. For example, professionals described that their ability to reach out to parents could be strengthened simply by having the tool available to offer parents during conversations or via websites. Likewise, Interplay could support and strengthen the work of professionals in promoting parental equality within the couple relationship by acting as a tool that professionals could use in their daily work to talk to parents about various topics. In this way, it could support professionals in getting closer to parents' thoughts and reflections and in their work in identifying individual needs for support among parents.

…issues that have been raised can be discussed with us, we can also know if we need to refer further and in what way we can support … (Midwife, Antenatal Care)

Similarly, results illustrated that professional competence could contribute to enhancing the benefits of Interplay. Professionals perceived that they could follow up on parents' use and support them in the reflections and conversations that have arisen, which may promote parents' understanding of their own situation and the joint parenthood. Likewise, professionals could, based on their competence, guide parents in their use and recommend parents to play specially selected chapters based on identified individual needs. In connection with this, reflections emerged that professionals should make individual assessments to rule out risks of potential conflicts increasing in couples with existing problems before recommending Interplay to parents. In contrast, perceptions emerged that professional assessments would not be necessary, this since Interplay may provide support for parents regardless of how they perceive their relationship. Professionals working in child health care expressed that Interplay has the potential to be included within several units that work with parental support and that it could thus constitute a support that follows parents during the transition to parenthood and the time after. For example, they described that parents could be introduced to Interplay by midwives in connection with antenatal care and that specialist nurses within the child health care could continue to follow up and support parents in their use of Interplay after the child is born.

…perhaps that you have to answer a certain number of questions until the next time … that you include it so that it becomes part of the caring meeting … (Midwife, Antenatal Care)

Similarly, professionals, based on their experiences of caring for parents, perceived that the couple relationship extends beyond the transition to parenthood and the first period after the birth of the child. They described that it could be valuable to develop and include Interplay as a support for more target groups and in more arenas to promote people's couple relationships as well as their shared parenting. For example, results revealed that it could be valuable to develop Interplay for parents who have older children aged 6–18 years and thus could constitute a support for their couple relationship and parenting that could be included in school health care. Similarly, it emerged that it could be valuable to develop Interplay for young people aged 13–25 years as a support in connection with them starting to establish their first couple relationships, which could be included in youth clinics and their work with sexual and reproductive health.

## 4 Discussion

The study aimed to explore how professionals perceive Interplay as a digital support tool for parents couple relationship and parenting. Through this, we wanted to increase knowledge about how professionals working with parental support perceive that digital support tools can be developed and function along with caring. The results showed that Interplay could function as a complement to existing care and available parental support, as Interplay could be a tool that parents could use flexibly in their everyday lives and that could engage both parents in the same process and support them in focusing on the couple relationship and shared parenting. This is similar to previous research on parents' perceptions, which has shown that Interplay can be a flexible support tool to use based on individual circumstances, which can facilitate for parents to make time for each other and focus on the couple relationship (19). The finding that Interplay could be a complement is confirmed by previous research that highlighted the need for parental support that includes both parents and focuses on the parents' relationship and emotional well-being (26). Similarly, the result can be understood in relation to previous research that has shown that interventions that highlight couple dynamics and facilitate shared parenting have been shown to strengthen individual well-being and the quality of parents' relationship (27).

The results showed that Interplay may encourage parents to communicate with each other and guide parents in how storytelling and listening can take place. This is interesting in relation to parents' perceptions that Interplay can act as a catalyst for communication within the couple relationship and support balanced conversations between parents (19). Similarly, the results showed that Interplay can provide a neutral basis for parents to touch on different topics that may be relevant to the parents' current life situation and the transition to parenthood. In order to promote parents' transition to parenthood, it can be understood as crucial to strengthen parents' awareness, knowledge, understanding and skills for the changes that parenthood may entail (28). Similarly, parents have expressed that Interplay can provoke reflections and broaden parents' views on expectations, role distribution and their individual strengths in relation to the couple relationship and parenthood (19). This suggests that Interplay may have the potential to support parents in communicating and preparing for the changes that parenthood may bring.

The transition to parenthood can be understood through learning processes linked to individual and collective processes. These can be described, for example, through individual learning in relation to internal changes and mental processes, as well as through interrelational learning in relation to one's partner and communication within the couple relationship (29). Similarly, communication has been shown to have an impact on commitment in the relationship and thus the quality of the couple's relationship (30). The quality of the parents' relationship can also affect their way of being parents, this can be understood by the fact that the quality of the relationship can affect the parents' ability to pay attention to and meet the child's needs, which thus affects the child's upbringing environment, safety and health (10). It can thus be understood that Interplay's potential to promote parents' communication with each other, in the long term, may be valuable for the entire family.

Current study created space for professionals to express their perceptions of Interplay as a digital support tool for parent couple relationship and shared parenting based on their experiences of supporting and caring for parents. Whereby introduction of new tools has been shown to be significantly facilitated if professionals working within the context are involved in the development and implementation of the tool (31). The results showed that it could be valuable to include Interplay in professional care and that it could be combined with professional competence, which in synergy could reinforce each other and lead to enhanced parental support. Nevertheless, further research is required to understand whether this is indeed a phenomenon that holds true within the care of parents. Interplay seems to have the potential to contribute to continuity and be included within different units that work with parental support. Whereby it could constitute a support that follows parents from pregnancy to the time after the child's birth. This can be understood in line with previous research that has highlighted that integrating digital forms of support together with physical visits can contribute to a holistic approach (32). Similarly, a shared vision of care between different units can promote both person-centered care and parental participation in care (33).

By including Interplay in future care, professionals perceived that it may support them and facilitate their work in connection with parental support. This, as it may promote the opportunities for professionals to reach parents with other forms of parental support that include both parents in a couple relationship. Previous research confirms the importance of developing and including digital forms of support within healthcare to meet parents' needs (34). Current result showed that Interplay also may function as a support for professionals in their daily work, this since it may function as a tool that professionals could use to identify parents' individual needs for support in connection with becoming parents. For example, it emerged that professionals could follow up on the thoughts and reflections that have arisen among parents in connection with their use of Interplay and that professionals, through their expertise could identify parents' individual needs in relation to Interplay. This is confirmed by previous research that highlighted that professional support needs to meet individual needs to a greater extent (35) and that individual needs for support need to be met in order for care to be caring (4). However, it has been shown that parents' needs for follow-up in connection with the use of Interplay may vary, with some parents perceiving it as valuable if professionals followed up on their use while others prefer to use Interplay on their own (19). This suggests that future inclusion of Interplay requires professionals to be responsive and adapt support to individual needs in connection with parents' use of Interplay.

Similarly, the results showed that professionals through their expertise may enhance the benefits of Interplay by recommending parents to play specifically selected chapters based on needs that professionals may identify in connection with caregiving. This is confirmed by previous research that has highlighted that it is crucial for the transition to parenthood that support is adapted to parents' individual needs (28). The results showed that professionals perceive that parents may have varied conditions for using Interplay, for example based on access to a mobile phone and knowledge of the Swedish language. Previous research has shown that digital forms of support can risk unequal care for parents who do not use it, this should be taken into account when including digital forms of support in care in order not to risk worsening health inequalities (36). For example, a future implementation would mean that Interplay needs to be developed to be available in more languages.

In an increasingly digitalized world, healthcare has a responsibility to support people in their ability to navigate and use digital health tools, thereby increasing patient engagement, improving health outcomes, and addressing equity challenges (37). Despite this, research on how digital forms of support can be integrated into healthcare is limited (38). Current study can be understood as a contribution to professionals' perceptions of how a digital support tool such as Interplay can be included in care and function as parental support. However, it should be noted that multiple perspectives are necessary in order for needs to be met in connection with the development and implementation of digital tools in healthcare (39). Thus, it can be understood that a future implementation of Interplay in healthcare would require additional perspectives and parallel research to continue studying challenges that may arise.

Although the current study contributes with valuable knowledge about professionals' perceptions of Interplay as a digital support tool for parents' relationship and parenting, limitations can be seen. To conduct international research on Interplay, it is necessary for Interplay to be translated and localized to more languages and settings, as the existing version is currently only available in Swedish format and a Swedish cultural setting. Participants were recruited through convenience sampling, which can be seen as preferable when recruiting for specific professions (24). Participants working with parental support from both antenatal care, child health care and family counselling with varying ages and experience in the profession were included, which increases the transferability to similar context (40). However, it should be taken into account that there may be differences within both health systems and cultures in the context of parenthood. In connection with the data collection, the COVID-19 pandemic occurred. This meant that the trivia questions in Interplay were updated on one occasion to keep the questions current. In addition, two interviews were conducted after the pandemic. It can be seen as a limitation that participants have touched on varying questions in Interplay and that the interview sessions span over a period of time. On the other hand, COVID-19 has led to expanded digital working methods in healthcare and since no differences emerged, it can be seen as a strength that the participants' perceptions are consistent in parallel with the previous pandemic and the ongoing development both in healthcare and digitally.

In the current study, 15 professionals used Interplay with a partner of their choice. This means that perceptions may differ from a future implementation and that further research on implementation is required. The professionals were interviewed until the authors considered that no new perceptions had emerged. Since data collection in qualitative research is stopped when all questions have been thoroughly explored and nothing new emerges in subsequent interviews (41), it requires the interviewer to be open and responsive to what emerges (21), a process that can be influenced by the interviewer's experience with interview situations (41). In current study, all interviews were conducted by two researchers with extensive experience with interview situations, which can be seen as a strength of the study. The results are presented with accompanying quotes, which can be seen to strengthen the study's analysis and thereby its credibility. The central issue for credibility in phenomenography can be understood to be the relationship between the empirical evidence and the categories (21). However, it should be noted that it is possible that other perceptions may exist, as people perceive phenomena in different ways (40). Based on the current study and previous research on parents' perceptions (19), Interplay is considered ready to be implemented in healthcare. However, it is important for future implementation that parallel research is conducted to study how professionals and parents experience digital support tools implemented in professional support and care for parents couple relationship and parenting.

## 5 Reflexivity statement

The authors' relationships to Interplay have varied based on their contributions to the current study. Within the framework of the larger research project, CB and HE have translated Interplay into Swedish and Swedish context. Technical development work has been carried out by HE, while AH and CB have updated the questions in Interplay. In connection with the study, all authors have continuously reflected together to manage personal, interpersonal, methodological and contextual processes. All authors had an open approach related to the fact that Interplay had not previously been studied, and that the app was studied in a Swedish context for expectant parents. In connection with interviews, the participants were informed about Interplay's background and the purpose of the study. The semi-structured interviews were conducted by a midwife (CB) and a specialist nurse (ML), the interviewers had experience from working in healthcare but did not work with parental support during the interviews. The authors are aware of the predominantly positive results, some of the authors have been involved in the development of Interplay, which may have influenced the interpretation of the data. This has been addressed through ongoing discussion within the author group, including the authors who were not involved in the development of Interplay (FL, ML), which may have balanced the results. However, more research is needed to study Interplay in a broader context.

## 6 Conclusion

Current study contributes with valuable knowledge about how digital support tools can be used and function as parental support. According to professionals, Interplay is perceived to have the potential to be a modern complementary digital tool that parents can use in a flexible and enjoyable way in their everyday lives to communicate and work with their relationship and parenting. However, professionals perceive that it should be taken into account that parents may have different conditions and needs. Interplay could be integrated within different care units such as antenatal care and child health care. In this way, professionals perceive that Interplay could be combined with professional competence and follow parents during the transition, which in turn can lead to enhanced parental support.

## Data Availability

The original contributions presented in the study are included in the article, further inquiries can be directed to the corresponding author.
