# Supplementary material for: Swedish health professional's perceptions of Interplay, a digital support tool for parents' couple relationship and parenting
Source: Front Digit Health. 2026 Aug 5;8:1912924. doi: 10.3389/fdgth.2026.1912924 (PMC13486215; doi:10.3389/fdgth.2026.1912924)
Supplement: Supplementary file 1 [file Datasheet1.pdf]

Additional file 1.
(Translated from Swedish and slightly edited for clarity in English)

Background questions:

1) What is your profession?
2) How old are you?
3) How many years of experience do you have in your profession?

Interview guide:

1) What are your experiences with parents using digital support?
2) What experience do you have in offering parents access to digital support?
3) How were your experiences using Interplay?
4) Was it easy to understand Interplay? Tell me more.
5) Was there any part of Interplay that was difficult to understand? Tell me more.
6) Which parts of Interplay did you appreciate more than others? Which parts and why those particular parts?
7) How appealing did you find Interplay?
8) How credible did you find the information in Interplay?
9) How did you perceive the questions in the game?
10) How appealing did you perceive Interplay?
11) How credible did you perceive the information in Interplay?
12) Do you see any risks and benefits of parents having access to Interplay?
13) What are your thoughts on introducing Interplay to parents?

Examples of follow-up questions:

How do you mean?

Can you tell me more?

Can you give an example?

How did it feel to you?
